# Draft Genome Sequences of the Fish Pathogen *Vibrio harveyi* Strains VH2 and VH5

**DOI:** 10.1128/genomeA.01062-15

**Published:** 2015-09-17

**Authors:** Daniel Castillo, Paul D’Alvise, Mathias Middelboe, Lone Gram, Siyang Liu, Panos G. Kalatzis, Constantina Kokkari, Pantelis Katharios

**Affiliations:** a Marine Biological Section, University of Copenhagen, Copenhagen, Denmark; b Department of Systems Biology, Technical University of Denmark, Copenhagen, Denmark; c BGI Europe A/S, Copenhagen, Denmark; d Institute of Marine Biology, Biotechnology and Aquaculture, Hellenic Centre for Marine Research, Former American Base of Gournes, Heraklion, Crete, Greece

## Abstract

*Vibrio harveyi* is an important marine pathogen that is responsible for vibriosis outbreaks in cultured fish and invertebrates worldwide. Here, we announce the draft genome sequences of *V. harveyi* strains VH2 and VH5, isolated from farmed juvenile *Seriola dumerili* during outbreaks of vibriosis in Crete, Greece.

## GENOME ANNOUNCEMENT

*Vibrio harveyi* is a marine bacterium that can be pathogenic to a wide range of organisms, especially fish and invertebrates (1, 2). The bacterium can cause mass mortalities in aquaculture species with significant economic impact for the industry. Several fish species can be infected by *V. harveyi*, including gilthead sea bream, European sea bass, common dentex, and Senegalese sole (3, 4). The virulence determinants of the pathogen have not been fully elucidated, however, and it has been suggested that they include biofilm formation, extracellular products, and quorum-sensing mechanisms (1). Here, we report the draft genomes sequences of *Vibrio harveyi* strains VH2 and VH5, which were isolated from juvenile *Seriola dumerili* during a vibriosis outbreak in Crete, Greece.

*V. harveyi* strains VH2 and VH5 were grown overnight at 22°C with agitation in Luria broth (MO BIO, no. 12106-05) supplemented with 1.7% NaCl. Genomic DNA was extracted using the QIAamp DNA miniKit (QIAGEN) according to the manufacturer’s protocol. A sequencing library was prepared using the Illumina HiSeq platform (BGI, China) with paired-end read sizes of 100 bp. A total of 10,356,788 paired-end reads for strain VH2 and 9,876,345 paired-end reads for strain VH5 were used for *de novo* assembly in Geneious version 7.1.7 (5). Short and low-coverage contigs were filtered out, resulting in a set of 107 contigs with an average coverage of 99× (*N*_50_, 110 Kbp), and 121 contigs with coverage of 105× (*N*_50_, 101 Kbp) for *V. harveyi* strains VH2 and VH5, respectively. Annotation was performed by the NCBI Prokaryotic Genome Automatic Annotation Pipeline (PGAAP) (6). Additionally, the genomes were analyzed on the Rapid Annotation using Subsystems Technology (RAST) server (7). Genome comparison was achieved using Mauve version 2.4.0 (8). Acquired antibiotic resistance genes were identified using ResFinder version 2.1 (9), virulence factors by VirulenceFinder version 1.2 (10), and prophage-related sequences using PHAST (11).

The final assembly for *V. harveyi* strain VH2 had a total length of 5,685,755 bp and a G+C content of 45%. Genome annotation resulted in 5,079 coding sequences (CDSs), 33 tRNAs, 38 pseudogenes, and 3 rRNAs. *V. harveyi* strain VH5 had a total length of 5,916,358 bp, and a G+C content of 44.9%. Genome annotation resulted in 5,254 CDSs, 93 tRNAs, 56 pseudogenes, and 3 rRNAs. Genome comparison showed that *V. harveyi* strain VH2 possessed 9 specific genomic islands between 6 and 26 Kbp and that strain VH5 contained 12 genomic islands between 7 and 21 Kbp. For both strains, putative virulence factors were identified with functions such as accessory colonization factors, bile hydrolysis, and production of colicin V. Genes with antibiotic resistance to fluoroquinolones, tetracycline, and colicin E2 were found. Interestingly, strain VH5 presented accessory cholera toxin and zona occludens cholera toxin. No prophage-related elements were detected.

Thus, these genome sequences can facilitate the future comprehensive comparison and phylogenetic analyses aiming toward the development of rational diagnostic and efficient control schemes of this fish pathogen.

### Nucleotide sequence accession numbers.

The draft genome sequence of *V. harveyi* strain VH2 can be accessed under the GenBank accession number LGYS00000000 and *V. harveyi* strain VH5 under the accession number LGYT00000000. The versions described in this paper are the first versions, LGYS10000000 and LGYT10000000.
